# PB.11: Identifying women at high risk of developing breast cancer: implications of adjusting for inter-observer variability in visual analogue scale assessment of percentage breast density

**DOI:** 10.1186/bcr3512

**Published:** 2013-11-08

**Authors:** JC Sergeant, M Sperrin, L Bardwell, I Buchan, DG Evans, A Howell, SM Astley

**Affiliations:** 1 Centre for Imaging Sciences, Institute of Population Health, University of Manchester, UK; 2 Centre for Health Informatics, Institute of Population Health, University of Manchester, UK; 3 Department of Mathematics and Statistics, University of Lancaster, UK; 4 Nightingale Centre and Genesis Prevention Centre, University Hospital of South Manchester, UK

## Introduction

Breast density is a well-established risk factor for breast cancer, with assessment of percentage density via a visual analogue scale (VAS) a practical method of measurement strongly associated with risk. We present a method to adjust for inter-observer differences in VAS density estimates and examine the effect of adjustment on the classification of women at high risk of developing breast cancer.

## Methods

A two-stage method is used to make estimates by different observers comparable. Results from all observers are transformed onto the same distribution, then differences in case mix are accounted for. We applied our approach to 13 experienced readers assessing 13,694 screening mammograms from a large clinical study where women are categorised as high risk if they have a 5 to 8% 10-year risk computed by a validated risk model and their breast density is in the top decile of the study population.

## Results

A total of 1,125 women were assessed as having a 10-year risk of 5 to 8%. Initially 126 of these were also high density, therefore classified as high risk, rising to 147 after density adjustment. After adjustment, 35 women were reclassified from nonhigh to high risk (3.5% of those initially nonhigh risk) and 14 women were reclassified from high to nonhigh risk (11.1% of those initially high risk).

## Conclusion

Adjusting VAS estimates of breast density for inter-observer variation substantially affected which women were classified as high risk of developing breast cancer. If VAS assessment of density is to be used in risk assessment to inform screening strategies, adjustment must be considered.

